# NCOA4: An Immunomodulation-Related Prognostic Biomarker in Colon Adenocarcinoma and Pan-Cancer

**DOI:** 10.1155/2022/5242437

**Published:** 2022-06-16

**Authors:** Chenzheng Gu, Wenjing Chang, Junlu Wu, Yiwen Yao, Gege Liu, Yi Yuan, Wenqiang Quan, Zujun Sun, Anquan Shang, Dong Li

**Affiliations:** ^1^Department of Laboratory Medicine, Shanghai Tongji Hospital, School of Medicine, Tongji University, Shanghai 200065, China; ^2^Department of Internal Medicine V-Pulmonology, Allergology, Respiratory Intensive Care Medicine, Saarland University Hospital, Homburg 66424, Germany

## Abstract

Treatment of cancer in humans requires a thorough understanding of the multiple pathways by which it develops. Recent studies suggest that nuclear receptor coactivator 4 (NCOA4) may be a predictive biomarker for renal cancer. In the present work, TCGA, GEPIA, and several bioinformatics approaches were used to analyze the NCOA4 expression patterns, prognostic relevance, and association between NCOA4 and clinicopathological features and immune cell infiltration. We investigated NCOA4 expression in malignancies. Low NCOA4 expression was associated with poor overall survival in individuals with malignancies such as cholangiocarcinoma, colon adenocarcinoma, and clear cell renal carcinoma. We also analyzed NCOA4 DNA methylation in normal and primary tumor tissues and investigated possible functional pathways underlying NCOA4-mediated oncogenesis. In conclusion, downregulation of NCOA4 is associated with poor prognosis, and NCOA4 may be a predictive biomarker for COAD.

## 1. Introduction

Worldwide, cancer is the second leading cause of death, with mortality rates far exceeding those of human immunodeficiency virus/acquired immunodeficiency syndrome, tuberculosis, and malaria combined [1]. Cancer incidence and mortality are increasing at an alarming rate worldwide. It is difficult to pinpoint the exact reasons for this, although they are related both to population aging and growth and to changes in the incidence and distribution of the major risk factors for cancer, some of which are associated with social and economic development. Treatment options for cancer available today include surgical procedures as well as chemotherapy, radiation, targeted therapy, and immunotherapy. Due to the drawbacks of standard treatment, such as intolerance and toxicity, clinical outcomes have remained inadequate in most cases. To better tailor therapy, susceptibility genes need to be incorporated into future targeted and tailored cancer therapies [2]. It is critical to advance our knowledge of carcinogenesis through the discovery and characterization of novel cancer-spanning genes to deepen our understanding of the disease. The availability of freely accessible cancer genome resources such as TCGA (The Cancer Genome Atlas) [3], GEPIA (Gene Expression Profiling Interactive Research) [4], and TIMER (Tumor Immune Estimation Resource) [5] may now enable a more comprehensive and systematic analysis of cancer genes for a variety of tumors.

Nuclear receptor coactivator 4 (NCOA4), also known as androgen receptor-associated protein 70 (ARA70), was originally discovered to be a component of the RET-fused gene expressed in a subset of papillary thyroid carcinomas [6]. Autophagy is a type of selective autophagy regulated by NCOA4, an autophagy cargo receptor that binds to FTH1 (ferritin heavy chain 1) on the phagophore and transports it to the lysosome for degradation, releasing iron for systemic physiological needs [7]. Arginine on the surface of FTH1 has been shown to specifically bind NCOA4, which can then fuse with a lysosome via nascent autophagosomes, leading to acceleration of ferroptosis cell death. In cancer cells, NCOA4 is a critical regulator of ferroptosis, and mutations in NCOA4 can lead to decreased ferroptosis of tumor cells by reducing intracellular free iron accumulation, glutathione synthesis, and reactive oxygen species, among other effects (ROS). It has been postulated that ferroptosis may be a potential therapeutic target for cancer because cancer cells have higher iron levels and are more susceptible to ferroptosis activation. In the future, it may also be possible to treat a number of ferroptosis-associated diseases by modulating ferroptosis. Thus, a more comprehensive understanding of the genes associated with ferroptosis could be much more helpful in developing personalized treatments. Given the already known role of NCOA4 in ferritin degradation, targeting the COPZ1/NCOA4/FTH1 axis may be a promising therapeutic strategy for the treatment of human glioblastoma (GBM) [8]. NCOA4 has the potential to play a critical role in cancer development and progression, as it is thought to be involved in tumor growth and metastasis spread. NCOA4 has only been studied in a small number of cancers. It is therefore critical to better understand the regulatory functions and molecular processes of NCOA4 in the cancer database in order to develop innovative therapeutic approaches for cancer.

The role of NCOA4 in prognosis and the immune response was investigated using a number of different datasets, including the TCGA, UALCAN, Kaplan–Meier Plotter, TIMER, and cBioPortal. Additionally, we included factors such as survival status, genetic alterations such as DNA methylation, and critical cellular downstream pathways in our analysis of NCOA4 expression levels among tumor types. A molecular mechanism through which NCOA4 may contribute to the oncogenesis of a variety of human malignancies has been elucidated by our extensive and systematic pan-cancer research efforts.

## 2. Materials and Methods

### 2.1. NCOA4 Expression Pattern in Human Pan-Cancer

The data from TCGA (https://genomecancer.ucsc.edu/) were used to explore the dysregulation of NCOA4 expression between various kinds of cancer and normal tissues. The TCGA database was merged with GTEx (Genotype-Tissue Expression) [9] to gather RNA sequencing data and clinical features of patients with 33 different kinds of cancer [10]. Because the TCGA data for ACC, DLBC, LAML, LGG, OV, and UCS were insufficient, we used GEPIA2 (https://gepia2.cancer-pku.cn/#analysis) to generate box plots of the data using the usual log2-fold change cut-off of 1, *p* value cut-off of 0.01, and combined the TCGA and GTEx databases for a comprehensive analysis. The UALCAN tool (https://ualcan.path.uab.edu/analysisprot.html) [11] was used to determine the NCOA4 protein expression levels in distinct cancer types. The level of expression of NCOA4 total protein was determined in tumor and normal tissues.

### 2.2. Immunohistochemistry (IHC)

To assess changes in NCOA4 protein expression levels, we used the online HPA (Human Protein Atlas) (https://www.proteinatlas.org/) [12]. NCOA4 IHC pictures were retrieved in a variety of tissues, including BRCA, COAD, KIRC, LIHC, LUAD, and THCA.

The method of IHC referred to the previous research [13, 14]. Fresh tissues from Tongji Hospital's gastrointestinal surgery were sliced into 4 um thick slices, and then immunostaining was performed. Dewaxing of paraffin slices was followed by antigen retrieval using a 10 mmol/L citrate buffer at a pH of 6.0. For 15 minutes, sections were treated with methanol containing 3% hydrogen peroxide. After washing with PBS, the sections were incubated for 30 minutes with blocking serum. The sections were then treated overnight at 4°C with an anti-NCOA4 antibody (Abmart, Shanghai, China). Secondary antibody incubation and staining were conducted according to the manufacturer's instructions using the EnVision®+ System-HRP (DAB) kit (Dako, Glostrup, Denmark). The nuclei were counterstained with hematoxylin. NCOA4 was discovered to be highly expressed in normal tissue, as shown by brown staining under a microscope.

### 2.3. Prognostic Analysis

Forest plots and Kaplan–Meier curves were used to illustrate the relationship between NCOA4 expression and patient prognosis in 33 cancer types, including OS (overall survival) and DSS (disease-specific survival). Univariate survival analysis was used to compute the HR (hazard ratios) and 95% CI (confidence intervals). We analyzed survival data, including OS and DFS, for all TCGA cancer types (Supplementary Table S1), with or without NCOA4 genetic mutation, using the cBioPortal program (https://www.cbioportal.org/).

### 2.4. Genetic Alteration Analysis

We collected data on NCOA4 alteration frequency, mutation type, mutated site information, and CNA (copy number alteration) across all TCGA cancers using cBioPortal (https://www.cbioportal.org/) [15]. The UALCAN database was used to compare the methylation status of NCOA4 in various malignancies to that in surrounding normal tissues. The Student's *t*-test was used to analyze the data, and a *p* value of 0.05 was considered statistically significant.

### 2.5. Correlation of the NCOA4 Expression with Immune Infiltration

TIMER tool was used to examine the connection between NCOA4 expression and immune infiltration in all TCGA tumors. Cancer-associated fibroblasts, neutrophils, and macrophages, including M0, M1, and M2, were chosen for comprehensive examination. Estimates were made using the TIMER, TIDE, CIBERSORT, CIBERSORT-ABS, QUANTISEQ, XCELL, MCPcounter, and EPIC algorithms.

### 2.6. NCOA4-Related Gene Enrichment Analysis

The STRING website (https://string-db.org/) [16] was utilized to investigate the protein-protein interaction (PPI) of NCOA4. Additionally, we establish significant criteria such as the meaning of the network's edge (“evidence”), the sources of active interaction (“experiments”), the minimum required interaction score (“low confidence (0.150)”), and the maximum number of interactors to export (“no more than 50 interactors”). Then, we present the experimental evidence for the interaction network of ten NCOA4-binding proteins. GEPIA2 was used to identify the top 100 NCOA4-related genes from all TCGA tumor and normal tissue datasets. NCOA4 expression was compared to the common genes identified by interaction analysis using Spearman's correlation. Calculated *p* values and correlation coefficients (*R*) are shown in the respective figure panels. The heatmap representation of the selected genes' expression profile includes the partial correlation coefficient (cor) across all cancer types and the *p* value for the parity-adjusted Spearman's rank correlation test. To conduct KEGG (Kyoto Encyclopedia of Genes and Genomes) pathway analysis, we combined and filtered the NCOA4-related genes. The enhanced routes were shown using the R packages “tidyr” and “ggplot2.” Statistical significance was defined as a *p* value of 0.05.

### 2.7. Statistical Analysis

It was utilized to determine the expression of NCOA4 in various tissues. We compared NCOA4 expression levels in tumor and normal tissues using the *t*-test. Kaplan–Meier curves were used to analyze the survival of patients with varying levels of NCOA4 expression. We employed univariate Cox regression analysis to compute the HR and 95% CI in survival analysis. All R packages were run using version 4.0.3 of the R programming language (https://www.r-project.org/), and statistical significance was considered as a *p* value of 0.05.

## 3. Results

### 3.1. Pan-Cancer Expression Landscape of NCOA4

**Figure 1:**
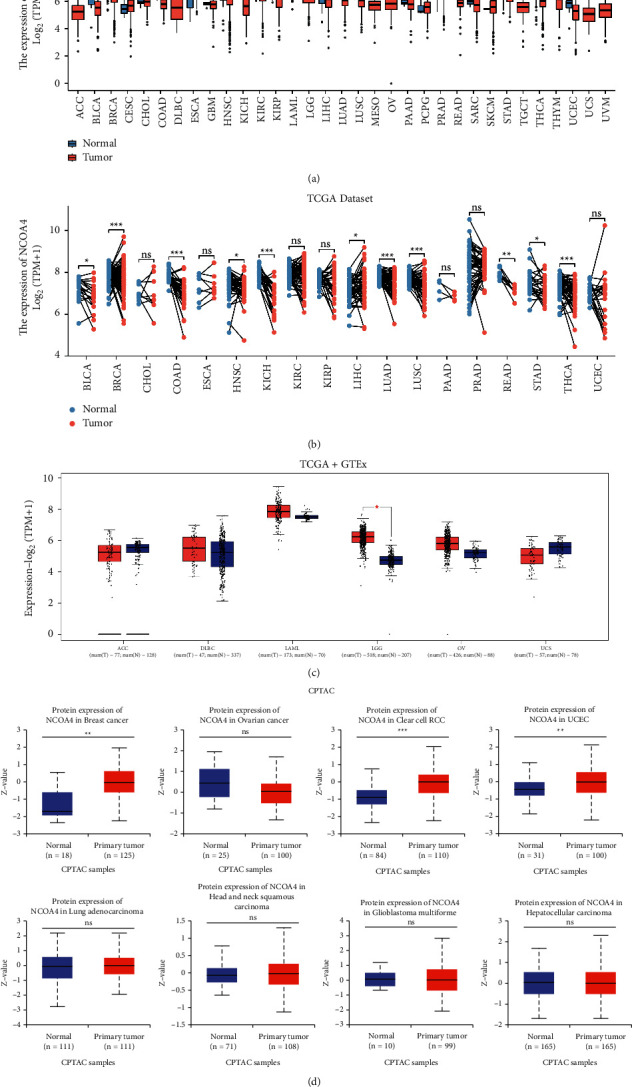
RNA and protein expression level of NCOA4 in human pan-cancer. (a) The expression level of NCOA4 in different human cancer types from the TCGA database. The red and blue boxes represent tumor tissues and unpaired normal tissues, respectively. (b) The expression level of NCOA4 in different human cancer types from the TCGA database. The red and blue boxes represent tumor tissues and paired normal tissues, respectively. (c) NCOA4 expression levels in ACC, DLBC, LAML, LGG, OV, and UCS were not shown by TCGA, instead of the GTEx database. (d) The protein level of NCOA4 in normal and tumor tissues, such as breast cancer, ovarian cancer, clear cell RCC, UCEC, LUAD, HNSC, GBM, and hepatocellular carcinoma, was analyzed by the CPTAC dataset. (ns: *p* > 0.05; ^*∗*^*p* < 0.05; ^*∗∗*^*p* < 0.01; ^*∗∗∗*^*p* < 0.001; ^*∗∗∗∗*^*p* < 0.0001).

**Figure 2:**
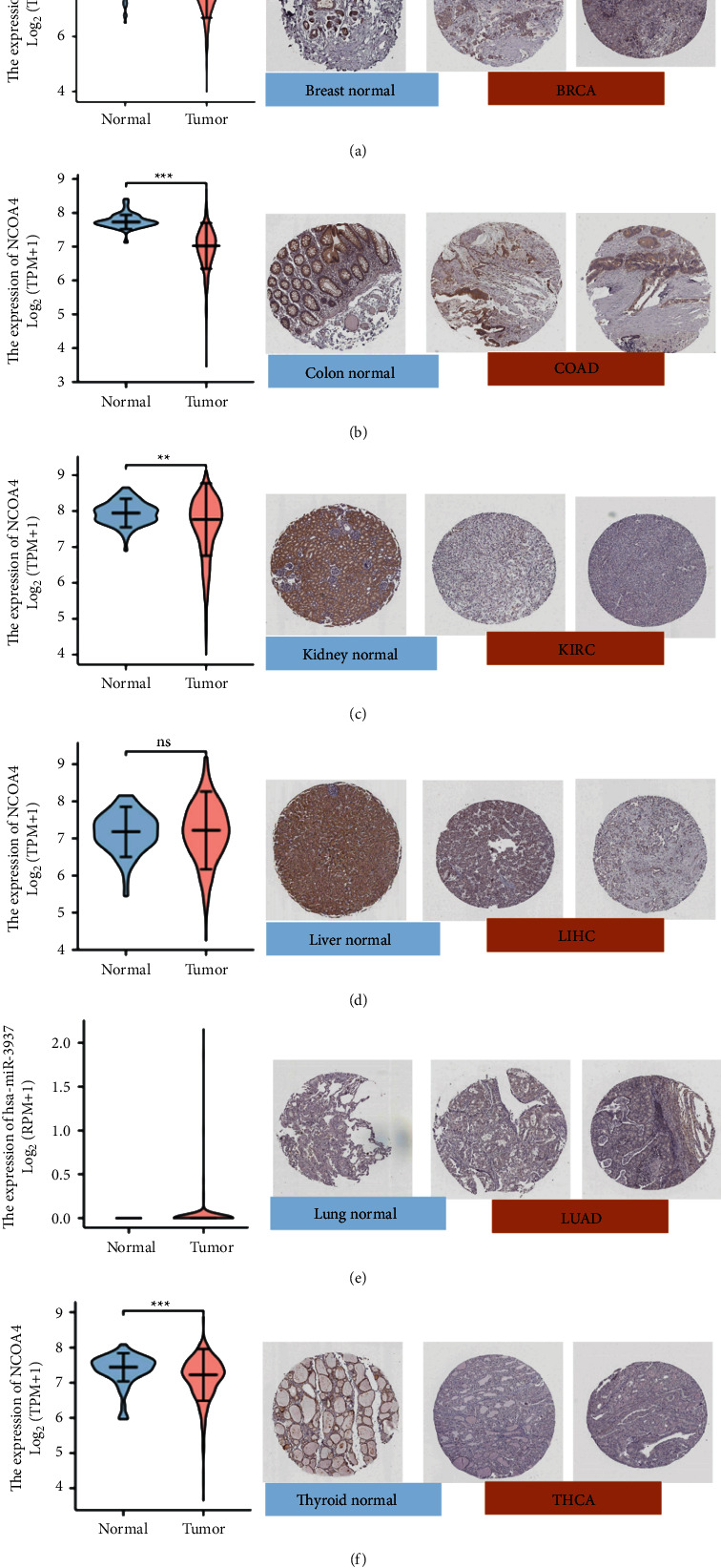
Comprehensive analysis of NCOA4 gene expression level in normal and tumor tissues by the TCGA (left) and immunohistochemistry images in normal and tumor (right) tissues by the HPA database. (a) Breast. (b) Colon. (c) Kidney. (d) Liver. (e) Lung. (f) Thyroid. (ns: *p* > 0.05; ^*∗*^*p* < 0.05; ^*∗∗*^*p* < 0.01; ^*∗∗∗*^*p* < 0.001; ^*∗∗∗∗*^*p* < 0.0001).

Our work intended to offer a complete characterization of the NCOA4 landscape in 33 different forms of cancer. NCOA4 expression was considerably increased in normal tissues of BLCA (*p* < 0.001), BRCA (*p* < 0.001), COAD (*p* < 0.001), HNSC (*p* < 0.05), KICH (*p* < 0.001), KIRC (*p* < 0.001), KIRP (*p* < 0.001), LUAD (*p* < 0.001), LUSC (*p* < 0.001), PRAD (*p* < 0.01), READ (*p* < 0.001), THCA (*p* < 0.001), and UCEC (*p* < 0.001) (Figure 1(a)). Furthermore, as shown in Figure 1(b), NCOA4 expression was considerably greater in normal tissues than in matched tumor tissues for BLCA (*p* < 0.05), BRCA (*p* < 0.001), COAD (*p* < 0.001), HNSC (*p* < 0.05), KICH (*p* < 0.001), LIHC (*p* < 0.05), LUAD (*p* < 0.001), LUSC (*p*=0.00018), READ (*p* < 0.01), STAD (*p* < 0.05), and THCA (*p* < 0.001). We further investigated the expression of NCOA4 in the GTEx database for some tumor types for which normal tissue data were not available in TCGA. However, we discovered a statistically significant difference between LGG and normal tissue (*p* < 0.05) (Figure 1(c)). Further analysis of the CPTAC dataset revealed that overall NCOA4 protein expression was significantly greater in breast cancer (*p* < 0.001), clear cell renal cell cancer (*p* < 0.001), and UCEC (*p* < 0.001) (Figure 1(d)). Additionally, we compared the HPA database's IHC results to the TCGA database's NCOA4 gene expression. Interestingly, only COAD, KIRC, LIHC, and THCA had consistent analytical findings between the two databases. Additionally, normal colon, kidney, lung, and thyroid tissues exhibited moderate to high IHC staining for NCOA4, but malignant tissues exhibited negative staining (Figures 2(a)–2(f)).

### 3.2. Pan-Cancer Analysis of the Correlation between NCOA4 Expression and Clinicopathology

**Figure 3:**
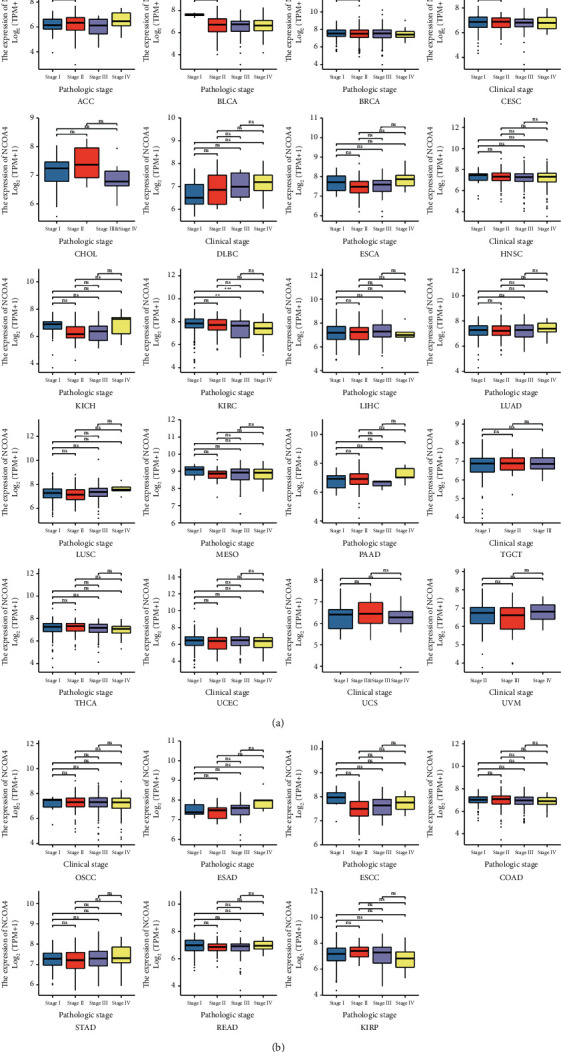
Correlation between the NCOA4 expression and clinical characteristics in pathological stage of various cancer types based on the TCGA dataset, including ACC, BLCA, BRCA, CESC, CHOL, DLBC, ESCA, HNSC, KICH, KIRC, LIHC, LUAD, LUSC, MESO, PAAD, TGCT, THCA, UCEC, UCS, UVM, OSCC, ESAD, ESCC, COAD, STAD, READ, and KIRP. (ns: *p* > 0.05; ^*∗*^*p* < 0.05; ^*∗∗*^*p* < 0.01; ^*∗∗∗*^*p* < 0.001; ^*∗∗∗∗*^*p* < 0.0001).

We examined NCOA4 expression in stage I, II, III, and IV pan-cancer patients to further study the association between NCOA4 expression and clinical features. The TCGA database analysis found that NCOA4 expression was significantly greater in the early stages of BLCA and KIRC (Figure 3). Additionally, we reported the relationship between the degree of NCOA4 expression and various clinicopathological characteristics, such as age, gender, T stage, N stage, M stage, and overall survival, in pan-cancer types (Supplementary Tables S2–S14).

### 3.3. Pan-Cancer Analysis of the Prognostic Value of NCOA4

**Figure 4:**
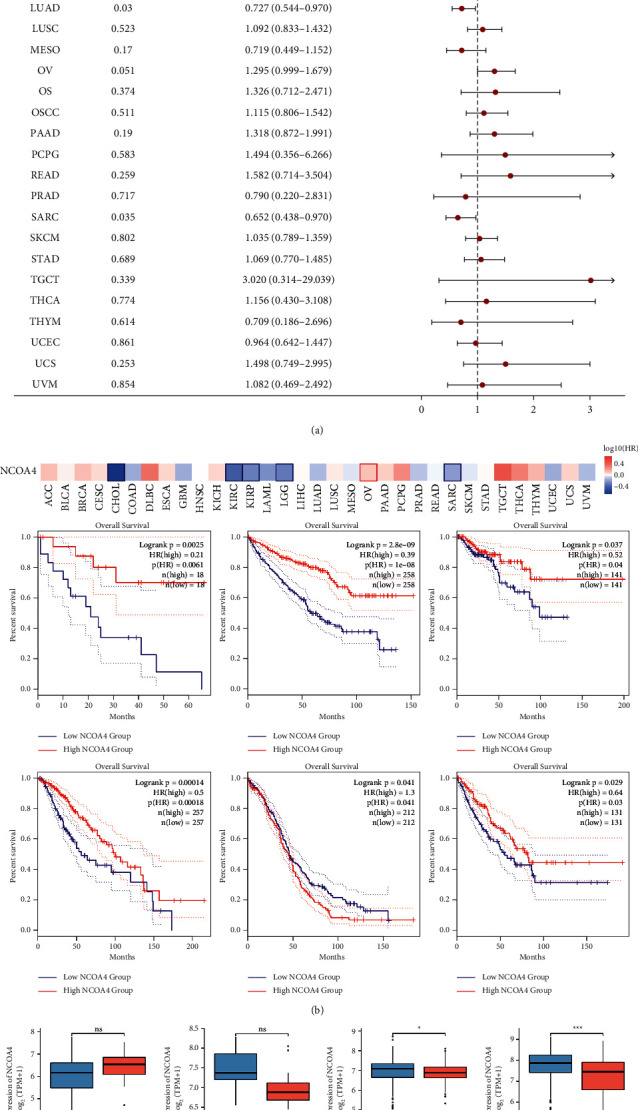
Relationship between the NCOA4 expression and the patients' overall survival in various cancer types. (a) The forest plot of hazard ratios of NCOA4 in human pan-cancer. (b) OS survival map of various cancer types. The significant results of Kaplan–Meier curves are listed. (c) The expression of NCOA4 in alive and dead individuals with cancer types.

**Figure 5:**
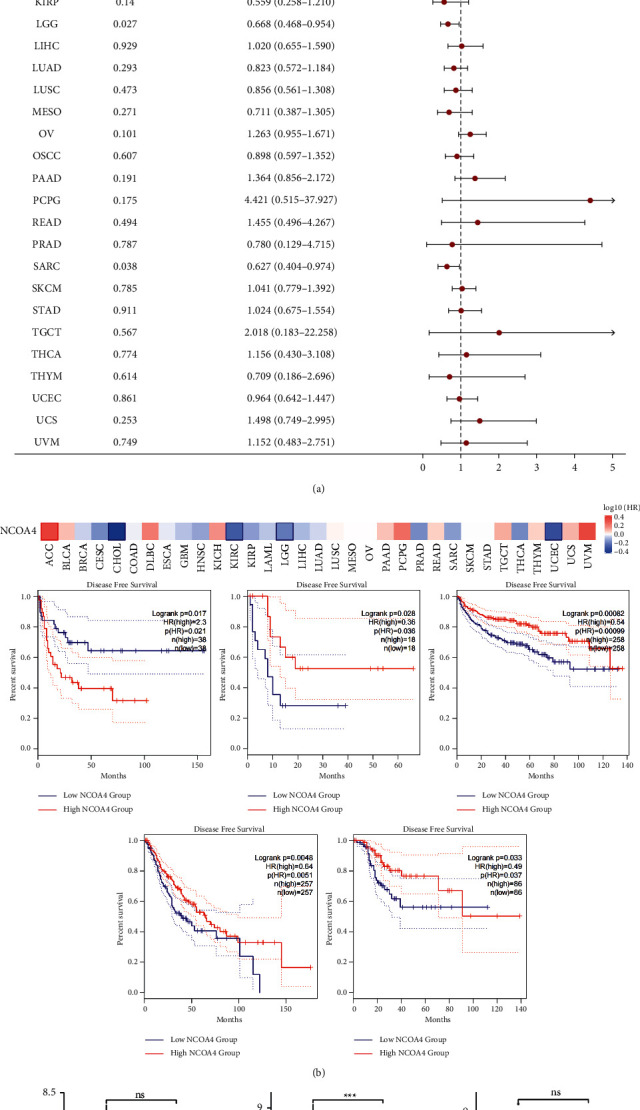
Relationship between the NCOA4 expression and the patients' disease-specific survival in various cancer types. (a) The forest plot of hazard ratios of NCOA4 in human pan-cancer. (b) DSS survival map of various cancer types. The significant results of Kaplan–Meier curves are listed. (c) The expression of NCOA4 in alive and deceased individuals with the aforementioned cancer types.

The purpose of this study was to determine the relationship between NCOA4 expression and prognosis and patient survival. We classified cancer patients according to their level of expression. We examined the connection between the NCOA4 expression and the prognosis of patients with pan-cancer using the TCGA database. OS and DSS were used as survival measures. In terms of OS, the Cox regression analysis of the 33 forms of cancer demonstrated a significant correlation between the NCOA4 expression level and seven types of cancer, namely, ACC, CHOL, COAD, KIRC, LGG, LUAD, and SARC. With the exception of ACC, decreased NCOA4 expression correlates with shorter overall survival in six kinds of cancer (Figure 4(a)). Additionally, we examined the distribution of NCOA4 expression levels and their connection to patient overall survival using the online database GEPIA2 (Figure 4(b)). The survival map and KM curves revealed that less NCOA4 expression in CHOL (*p*=0.0061), KIRC (*p* < 0.001), KIRP (*p*=0.575), LGG (*p*=0.00018), and SARC (*p*=0.03) led to unsatisfactory outcome. On the other hand, elevated NCOA4 expression in OV (*p*=0.041) resulted in a negative effect. Interestingly, the TCGA results for CHOL, KIRC, LGG, and SARC were congruent with the GEPIA2 database results. In addition to the TCGA data analysis, we examined the level of NCOA4 expression of alive and deceased patients with ACC, CHOL, COAD, KIRC, LGG, LUAD, and SARC. COAD had statistically significant differences (*p* < 0.05) and KIRC had a significant difference (*p* < 0.001) (Figure 4(c)). On the other hand, we used the same approaches to examine the link between NCOA4 expression and disease-free survival. The association between NCOA4 expression and DSS was discovered using Cox regression analysis of 33 different forms of cancer. Reduced NCOA4 expression correlates with shorter disease-free survival in four forms of cancer, namely, CHOL, KIRC, LGG, and SARC (Figure 5(a)). The survival map and KM curves demonstrated that decreased NCOA4 expression in CHOL (*p*=0.036), KIRC (*p*=0.00099), LGG (*p*=0.0051), and SARC (*p*=0.037) was associated with a less favorable result (Figure 5(b)). Additionally, we examined the expression of NCOA4 in alive and deceased individuals with the aforementioned cancer types. However, because the data for the DSS incident was insufficient, we gathered data from CHOL, KIRC, and SARC. In KIRC, there was a statistically significant difference (*p* < 0.01) (Figure 5(c)).

### 3.4. Validation of NCOA4 Expression and Prognostic Value in COAD

**Figure 6:**
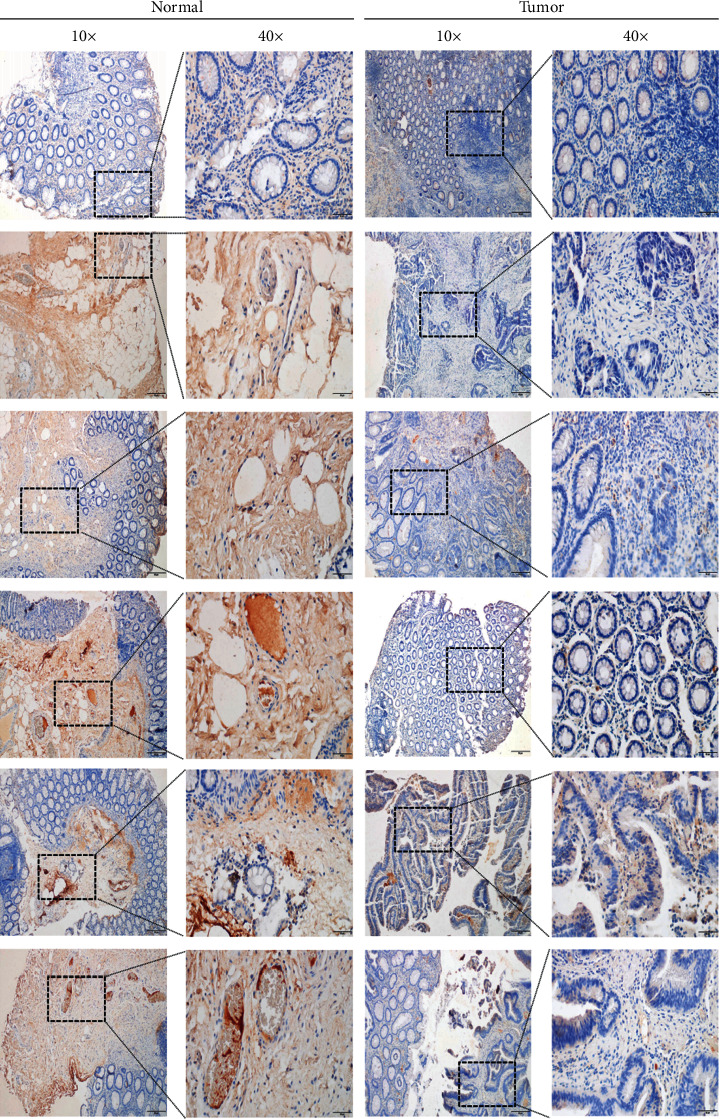
Analysis of the NCOA4 protein expression in COAD tissue by IHC. (a) The expression level of NCOA4 protein in COAD tumor tissue was lower than that in normal tissue. Brown (positive) and blue (counter) staining.

**Figure 7:**
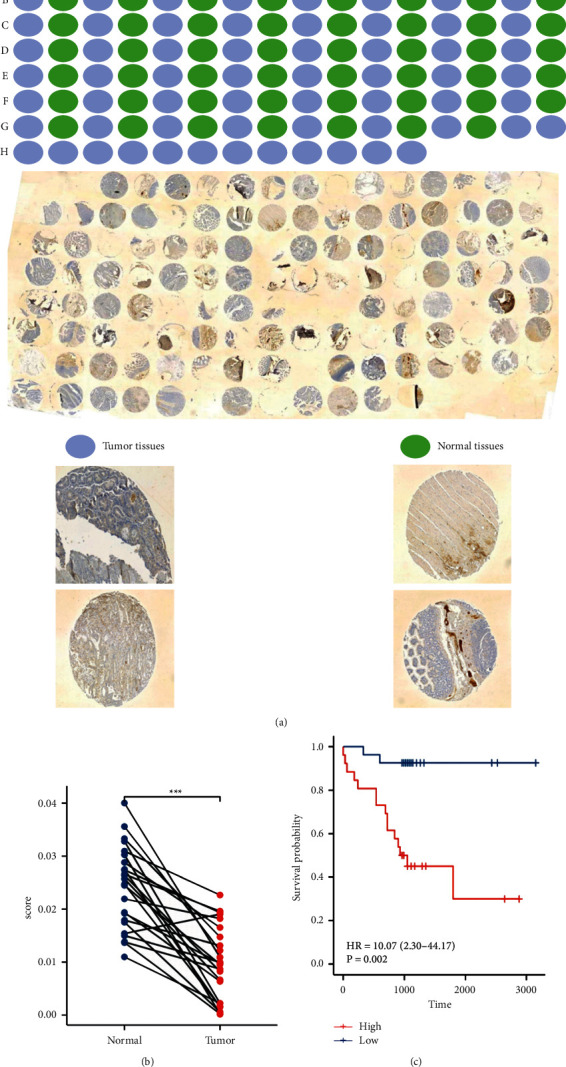
Analysis of the NCOA4 protein expression in COAD tissue microarrays by IHC. (a) The landscape of the COAD tissue microarrays. Blue dots represent tumor tissues and green dots represent normal tissues. (b) The expression level of NCOA4 in COAD. The red and blue boxes represent tumor tissues and paired normal tissues, respectively. (c) Kaplan–Meier curves of COAD patients. The red and blue curves represent low and high expression of NCOA4. (ns: *p* > 0.05; ^*∗*^*p* < 0.05; ^*∗∗*^*p* < 0.01; ^*∗∗∗*^*p* < 0.001; ^*∗∗∗∗*^*p* < 0.0001).

From the pan-cancer study results above, we find that NCOA4 gene expression in COAD and KIRC was consistent with protein expression as determined by IHC staining. Additionally, there were statistically significant associations between NCOA4 expression and OS in both cancer types. As a consequence, we chose the COAD for validation. Tongji Hospital obtained fresh tumor and peritumoral surrounding tissues from patients undergoing excision for localized COAD. The expression of the NCOA4 protein is decreased in COAD tissues (Figure 6). Additionally, we expanded the size of the tissue samples to verify the accuracy of the TCGA bioinformatics data. Shanghai Zhuoli Biotechnology Co. Ltd. (China) produced tissue microarrays with 40 sets of samples. Notably, the expression of NCOA4 protein was much greater in peritumoral neighboring tissues than in tumor tissues (Figures 7(a)and 7(b)). We defined the microarray landscape in terms of blue dots representing tumor tissues and green dots representing normal tissues. In these samples, the level of NCOA4 expression was highly associated with overall survival, and patients with low protein expression had a worse prognosis (*p*=0.002). (Figure 7(c)). This discovery established that low NCOA4 levels are related to worse clinical and prognostic outcomes.

### 3.5. Pan-Cancer Analysis of Genetic Alteration and Methylation of NCOA4

**Figure 8:**
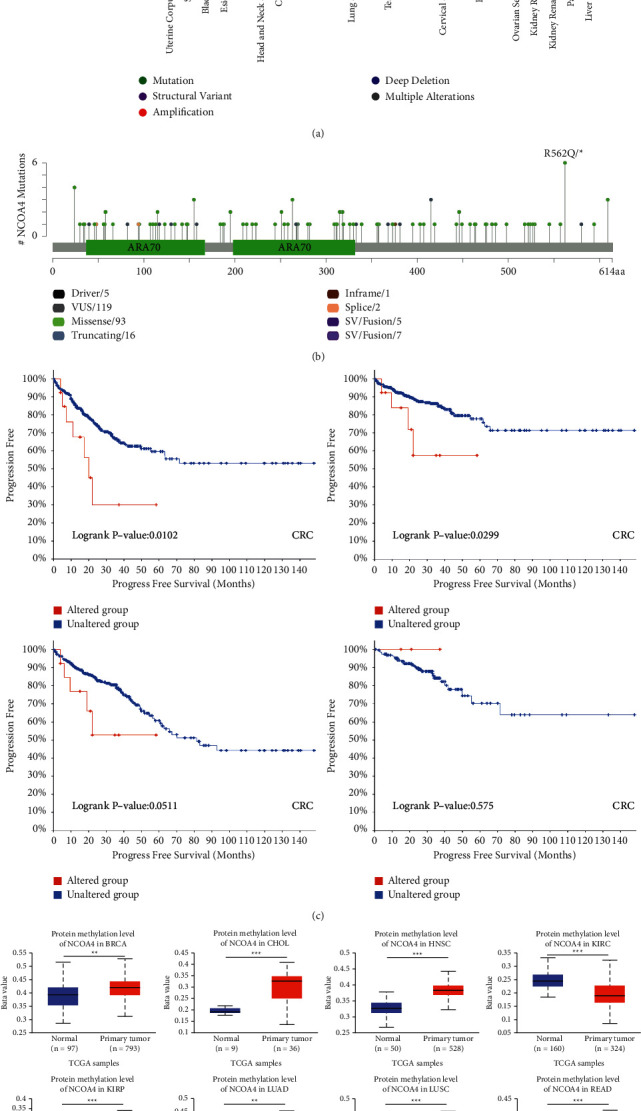
Mutation status of NCOA4 in TCGA tumors. (a) The alteration frequency with mutation type. (b) The mutation site of NCOA4. (c) Analysis of the relationship between mutation status and OS, DSS, disease-specific survival (DFS), and progression-free survival (PFS) of CRC, analyzed by cBioPortal tool. (d) Promoter methylation level of NCOA4 in pan-cancer. (ns: *p* > 0.05; ^*∗*^*p* < 0.05; ^*∗∗*^*p* < 0.01; ^*∗∗∗*^*p* < 0.001; ^*∗∗∗∗*^*p* < 0.0001).

Disruption of the epigenetic landscape occurs often in cancer, frequently as a result of genetic abnormalities in epigenetic regulatory genes [17]. Cancer develops as a result of an accumulation of epigenetic and genetic changes [18]. As a result, we sought to investigate the NCOA4 genetic variants using the cBioPortal (TCGA, Pan-Cancer Atlas). According to our findings, the modification frequency (>5%) with “mutation” of NCOA4 is higher in primary uterine cancers. Furthermore, the frequency of modifications (>5%) in NCOA4 “structural variation” and “amplification” in cholangiocarcinoma was the greatest kind (Figure 8(a)). The following step was to investigate the new mutations and their placement within NCOA4 (Figure 8(b)). The dominant mutation type for NCOA4 was identified as “Missense.” However, the online database does not provide information about the three-dimensional structure of the NCOA4 protein. We conducted a comprehensive review of these variants in diverse kinds of malignancies to determine whether there is an association between certain NCOA4 genetic variants and patient clinical survival prognosis. Patients with UCEC who had a genetic variant of NCOA4 had a better prognosis in terms of progression-free survival (*p*=0.0102), disease-specific survival (*p*=0.0299), but not overall survival (*p*=0.0511), or disease-free survival (*p*=0.575), when compared to patients who did not have a mutation of NCOA4 (Figure 8(c)). Genome-wide analyses of DNA methylation in a range of cancer types have revealed a high prevalence of cancer-associated methylation alterations.

DNA methylation changes occur in every kind of cancer, and more significantly, DNA methylation levels correlate with tumor aggressiveness in the majority of cancers [19]. As a result, we investigated the DNA methylation of NCOA4 using the UALCAN and TCGA datasets (Figure 8(d)). The significant increase in methylation of NCOA4 was shown in BRCA (*p* < 0.01), CHOL (*p* < 0.001), HNSC (*p* < 0.001), LUAD (*p* < 0.01), and LUSC (*p* < 0.001). On the contrary, we found a significant decrease in methylation of NCOA4 in KIRC (*p* < 0.001), KIRP (*p* < 0.001), READ (*p* < 0.001), TGCT (*p* < 0.001), THCA (*p* < 0.01), and UCEC (*p* < 0.001).

### 3.6. Pan-Cancer Analysis of the NCOA4 Expression and Immune Cell Infiltration

**Figure 9:**
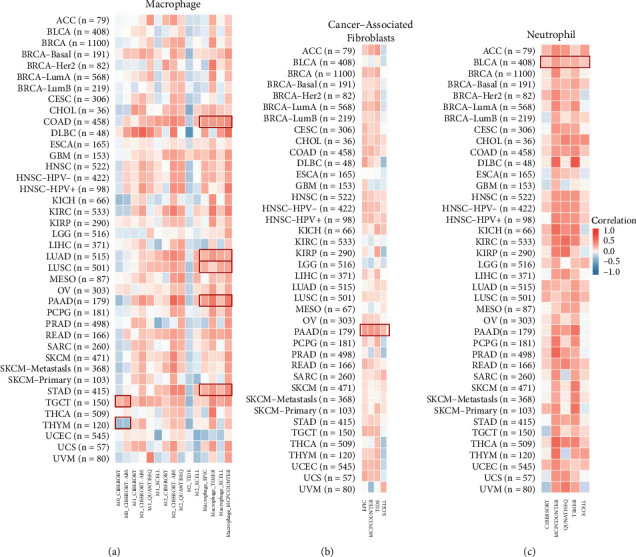
The correlation between NCOA4 expression and immune cell infiltration in TME. (a–c) TIMER, TIDE, CIBERSORT, CIBERSORT-ABS, QUANTISEQ, XCELL, MCPcounter, and EPIC algorithms were utilized to analyze the relationship between NCOA4 expression and macrophage, cancer-associated fibroblasts, and neutrophil across all tumor types in the TCGA database. The red color indicates a positive correlation (0-1), while the blue indicates a negative correlation (−1-0). *p* value <0.05 is considered statistically significant.

Lymphocytes invading tumors affect cancer patient survival. As a result, we examined the expression of NCOA4 in invading immune cells. Thus, we used the TIMER, CIBERSORT, CIBERSORT-ABS, TIDE, XCELL, MCPcounter, QUANTISEQ, and EPIC algorithms to investigate the relationship between immune cells infiltration and NCOA4 expression in several TCGA tumor types. Interestingly, we observed a negative association between the NCOA4 expression and the estimated macrophage M0 infiltration value for THYM and a positive correlation for TGCT. Additionally, we identified a strong connection between NCOA4 expression and predicted macrophage infiltration values for COAD, LUAD, LUSC, PAAD, and STAD (Figure 9(a)). NCOA4 expression and cancer-associated fibroblast infiltration for the PAAD also exhibited a positive link, whereas neutrophils and NCOA4 expression were shown to be positively correlated with BLCA tumors (Figures 9(b) and 9(c)).

### 3.7. Enrichment Analysis of NCOA4-Related Partners

**Figure 10:**
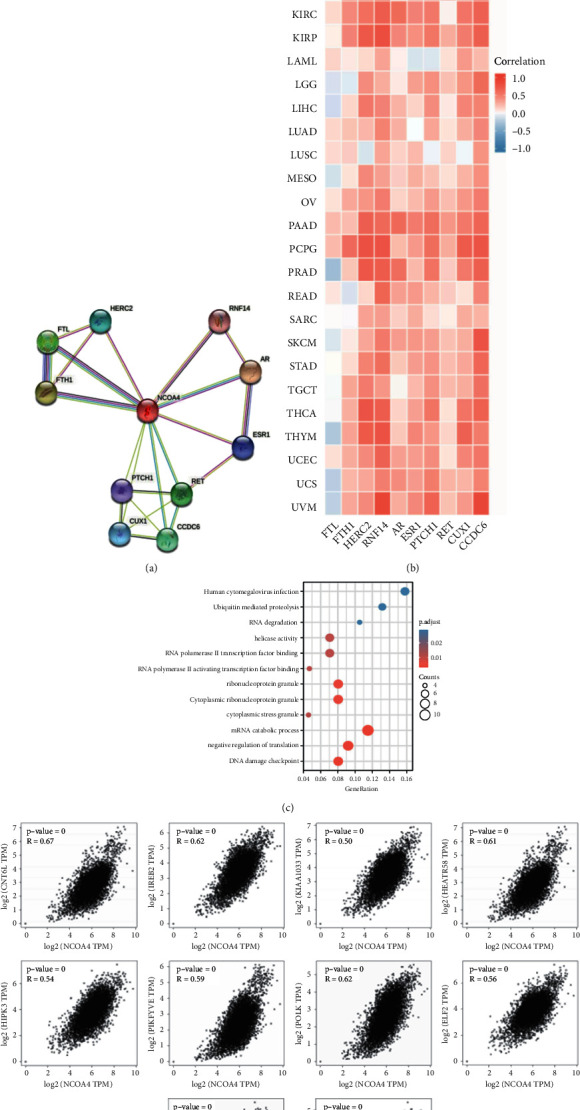
NCOA4-related genes enrichment and pathway analysis in pan-cancer. (a) Protein-protein interaction network was analyzed by STRING. (b) Heatmap representation of the expression correlation data between NCOA4 and FTL, FTH1, HERC2, RNF14, AR, ESR1, PTCH1, RET, CUX1, and CCDC6 in all TCGA tumors. The red color indicates a positive correlation (0-1), while the blue indicates a negative correlation (−1-0). (c) GO/KEGG pathway based on the top 100 NCOA4-related genes in the TCGA database. (d) Expression correlation between NCOA4 and the top 10 NCOA4-related genes (CNOT6L, REB2, KIAA1033, HEATR5B, HIPK3, PIKFYVE, POLK, ELF2, and FBXO38) in the TCGA database as determined by GEPIA2 tool. The *r* ranges from −1 to 1, where |*r*| ≥ 0.7 signifies a high positive correlation, 0.5 ≤ |*r*| < 0.7 is a moderate positive correlation, 0.3 ≤ |*r*| < 0.5 is a low positive correlation, and |*r*| < 0.3 is a negligible correlation.

Finally, to elucidate the molecular mechanism behind the NCOA4 gene's carcinogenesis and development, we used STRING techniques to screen for known NCOA4-interacting proteins, obtaining the top ten most relevant proteins, namely, FTL, FTH1, HERC2, RNF14, AR, ESR1, PTCH1, RET, CUX1, and CCDC6 (Figure 10(a)). Then, using the heatmap, we analyzed the association between the top 10 genes and NCOA4 in 33 different forms of cancer (Figure 10(b)). In KIRC, KIRP, PAAD, and PCPG, we discovered that these genes had a strong connection with NCOA4. Then, we utilized the GEPIA2 program to integrate all of the TCGA's tumor expression data and identified the top 100 genes that were significantly linked with NCOA4 expression. Using GEPIA2, we discovered that NCOA4 expression was positively linked with CNOT6L (*R* = 0.67), IREB2 (*R* = 0.62), KIAA1033 (*R* = 0.59), HEATR5B (*R* = 0.61), HIPK3 (*R* = 0.64), PIKFYVE (*R* = 0.59), POLK (*R* = 0.62), ELF2 (*R* = 0.56), and FBXO38 (*R* = 0.63), (Figure 10(c)). Finally, we integrated the two datasets to do enrichment analysis using GO and KEGG. The GO|KEGG pathway analysis indicated “DNA damage checkpoint,” “negative regulation of translation,” and “mRNA catabolic process” as top hits, suggesting that these pathways are involved in NCOA4's tumor pathogenesis-regulating function (Figure 10(d)). By comprehensive analysis of the above results, we found that the NCOA4 expression level was significantly associated with COAD and KIRC. We then explored the tradition hallmark pathways in COAD or KIRC, including tumor_inflammation_signature, cellular_response_to_hypoxia, tumor_proliferation_signature, EMT_markers, ECM-related_genes, angiogenesis, apoptosis, DNA_repair, G2M_checkpoint, inflammatory_response, PI3K_AKT_mTOR_pathway, P53_pathway, MYC_targets, TGFB, IL-10_anti-inflammatory_signaling_pathway, genes_upregulated_by_reactive_oxygen_species_(ROS), DNA_replication, collagen_formation, and degradation_of_ECM. In COAD samples, we found just the PI3K_AKT_mTOR_pathway had a low positive correlation (*r* = 0.36, *p* < 0.001) (Supplementary Figure S1). In KIRC, we found a similar result in the PI3K_AKT_mTOR_pathway (*r* = 0.38, *p* < 0.001). Additionally, we also found that there was a moderate negative correlation in the DNA_repair_pathway (*r* = −0.54, *p* < 0.001) (Supplementary Figure S2).

## 4. Discussion

Cancer accounts for a significant amount of the world's sickness burden, continuing to be a dangerous disease, with the number of cancer-related deaths increasing [20]. Patient survival has been significantly improved as a result of advancements in cancer diagnosis and treatment technology. It is possible to undertake a detailed study of prognosis at the pan-cancer level using high-throughput RNA expression data and clinical information from the TCGA Pan-Cancer project, which is supported by the National Cancer Institute. With the use of a pan-cancer investigation of approximately 10,000 original tumors spanning 33 various cancer types, we observed that low levels of NCOA4 in malignancies were linked with poor overall survival. The current work conducted a comprehensive analysis of NCOA4 expression in a pan-cancer dataset for the first time. The results from the analysis of 33 cancers' data from the TCGA revealed that NCOA4 was significantly downregulated in BLCA, BRCA, COAD, HNSC, KICH, KIRC, KIRP, LUAD, LUSC, PRAD, READ, THCA, and UCEC but upregulated in LGG, compared with the paracancerous and normal tissues (Figures 1(a) and 1(b)). NCOA4 expression levels may vary among tumors due to their diverse underlying functions and processes. Additionally, we discovered that decreased NCOA4 expression was associated with worse OS results in a variety of malignancies, including CHOL, COAD, KIRC, LGG, LUAD, and SARC (Figure 4(a)). Furthermore, we examined the distribution of NCOA4 expression levels and their connection to patient overall survival using the online database GEPIA2. The survival map and KM curves demonstrated that decreased NCOA4 expression in CHOL, KIRC, KIRP, LGG, and SARC results in poorer than desirable outcomes (Figure 4(b)). In addition to the TCGA data analysis, we examined the level of NCOA4 expression in alive and deceased patients with ACC, CHOL, COAD, KIRC, LGG, LUAD, and SARC. COAD and KIRC had statistically significant differences (Figure 4(c)). The TCGA results for CHOL, KIRC, LGG, and SARC were consistent with the GEPIA2 database results. We used the same methodology to examine the relationship between NCOA4 expression and DSS. Cox regression investigation of 33 forms of cancer found that decreased NCOA4 expression in four types of cancer, namely, CHOL, KIRC, LGG, and SARC, results in a shorter disease-free life (Figure 5(a)). The survival map and KM curves demonstrated that decreased NCOA4 expression results in an unfavorable outcome in CHOL, KIRC, LGG, and SARC (Figure 5(b)). These findings show that NCOA4 may be used as a biomarker for predicting tumor patient prognosis. Our study revealed numerous noteworthy findings, particularly at COAD and KIRC. Then, we used COAD as the validation cancer in order to investigate the relationship between the level of NCOA4 expression and the result of COAD (Figures 6 and 7). We discovered that a lower level of NCOA4 resulted in a poorer prognosis for COAD patients. This outcome was consistent with the TCGA dataset analysis. These findings provide a potential clinical biomarker for the prediction of COAD overall survival.

Cancer is a hazardous disease accounting for a major portion of the world's illness burden, with the number of cancer-related fatalities growing. It has been found that low expression of NCOA4, a ferritinophagy-related gene, correlates with decreased immune cell infiltration and impaired IFN-*γ* receptor signaling in clear cell renal carcinoma (ccRCC) [21]. Vandetanib may be a useful treatment strategy for CRC patients with NCOA4-RET fusion, according to the study in [22]. Hence, NCOA4 has potential as a novel marker to identify patients who may be eligible for ferroptosis-induction therapy or immunotherapy combined with it. As a consequence of breakthroughs in cancer diagnostic and treatment technologies, patient survival has increased dramatically in recent years. In addition, considering the clinical information from the TCGA Pan-Cancer project, which is financed by the National Cancer Institute, it is feasible to conduct a thorough assessment of prognosis at the pan-cancer level. We discovered that low levels of NCOA4 in malignancies were associated with poor overall survival after conducting a pan-cancer examination including almost 10,000 original tumors spanning 33 different cancer types in a single study.

As a result, we sought to investigate the NCOA4 genetic variants using the cBioPortal (TCGA, Pan-Cancer Atlas). According to our findings, the modification frequency (>5%) with “mutation” of NCOA4 is higher in primary uterine cancers. Moreover, the frequency of modifications (>5%) in NCOA4 “structural variation” and “amplification” in cholangiocarcinoma was the greatest kind (Figure 8(a)). The following step was to investigate the new mutations and their placement within NCOA4 (Figure 8(b)). The dominant mutation type for NCOA4 was identified as “Missense.” Because there is no evidence of NCOA4 being phosphorylated, we examined NCOA4's DNA methylation using the UALCAN and TCGA datasets (Figure 8(d)). DNA methylation, or the methylation of the carbon 5 atom of cytosines, occurs mostly in the CpG context in eukaryotes, but also in the CpHpG and CpHpH contexts in some cell types [23]. DNA methylation abnormalities have been linked to a range of human disorders, including cancer [24], and the most well-studied epigenetic mechanism of cancer is aberrant DNA methylation. Our research revealed a substantial increase in NCOA4 methylation in BRCA, CHOL, HNSC, LUAD, and LUSC. On the other hand, we observed a substantial reduction in NCOA4 methylation in KIRC, KIRP, READ, TGCT, THCA, and UCEC. Additionally, abnormal DNA methylation (hyper- or hypomethylation) may affect NCOA4 gene expression and consequently cancer processes. DNA hypermethylation occurs early and often in a variety of malignancies, and methylation of viral DNA has been proposed as a potential biomarker for cervical illness [25]. Since DNA methylation-mediated gene silence has been implicated in the pathogenesis of a variety of disorders, including cancer, targeting aberrant DNA methylation may one day be deemed a therapeutically useful technique for cancer therapy.

Particularly noteworthy is the observation that ferritinophagy increases ferroptosis [26–28]. Multiple studies have shown that autophagy contributes to ferroptosis during the early stages of the process by supplying accessible ferric iron through NCOA4-mediated ferritinophagy during the early stages of the process [29]. Additionally, since some oncogenic mutations render tumor cells very vulnerable to ferroptosis, triggering ferroptosis in tumor cells that are susceptible to ferroptosis may hold considerable therapeutic potential for ferroptosis-sensitive tumor cells [30]. However, there is still much that is unknown about the molecular pathways that support the therapeutic potential of ferroptosis regulation. It is impossible to understand tumor progression without understanding the tumor microenvironment, which is comprised of nontumor cells such as tumor-infiltrating immune cells (TILs), cancer-associated fibroblasts (CAFs), and endothelial cells. The absence of extracellular matrix (ECM) components results in the establishment of a highly dynamic tumor microenvironment (TME) with intricate interactions between multiple internal components that promote tumor growth and resistance to therapy. Because of this, integrating NCOA4 and TME research may provide novel insights into tumor treatment.

Finally, we conducted GO and KEGG enrichment analyses of NCOA4-interacting proteins identified using the STRING tools and GEPIA2 in pan-cancer samples to determine their functional significance. Using the GO|KEGG pathway analysis, we discovered that “DNA damage checkpoint,” “negative regulation of translation,” and “mRNA catabolic process” were the top hits, indicating that these pathways are engaged in NCOA4's role in tumor pathogenesis regulation. Our results may provide insight into the mechanism through which NCOA4 exerts its downstream effects.

## 5. Conclusions

In conclusion, our thorough pan-cancer analysis of NCOA4 demonstrated that it was related to a poor prognosis in individuals who had it downregulated. NCOA4 is an oncogene and a prognostic marker in COAD. Targeting NCOA4 may be a potential method for COAD therapy. Additional factors such as DNA methylation, immune cell infiltration, and NCOA4-interacting proteins may be of assistance in explaining the role of NCOA4 in the development of cancer from a variety of perspectives.

## Data Availability

The data supporting the findings of this study are available within the article. Further inquiries can be directed to the corresponding authors.

## Supplementary Materials

Supplementary Materials Figure S1. NCOA4-related gene hallmark enrichment pathway of COAD in the TCGA database. Figure S2. NCOA4-related gene hallmark enrichment pathway of KIRC in the TCGA database. Table S1. Abbreviations for cancer in TCGA. Table S2. The relationship between clinical features and NCOA4 expression levels in COAD. Table S3. The relationship between clinical features and NCOA4 expression levels in KIRC. Table S4. The relationship between clinical features and NCOA4 expression levels in LIHC. Table S5. The relationship between clinical features and NCOA4 expression levels in BRCA. Table S6. The relationship between clinical features and NCOA4 expression levels in CESC. Table S7. The relationship between clinical features and NCOA4 expression levels in CHOL. Table S8. The relationship between clinical features and NCOA4 expression levels in LAML. Table S9. The relationship between clinical features and NCOA4 expression levels in LGG. Table S10. The relationship between clinical features and NCOA4 expression levels in PRAD. Table S11. The relationship between clinical features and NCOA4 expression levels in LUAD. Table S12. The relationship between clinical features and NCOA4 expression levels in LUSC. Table S13. The relationship between clinical features and NCOA4 expression levels in SARC. Table S14. The relationship between clinical features and NCOA4 expression levels in SKCM.
