# Supplementary material for: NCOA4: An Immunomodulation-Related Prognostic Biomarker in Colon Adenocarcinoma and Pan-Cancer
Source: J Oncol. 2022 Jun 16;2022:5242437. doi: 10.1155/2022/5242437 (PMC9225891; doi:10.1155/2022/5242437)
Supplement: Supplementary Materials — Figure S1. NCOA4-related gene hallmark enrichment pathway of COAD in the TCGA database. Figure S2. NCOA4-related gene hallmark enrichment pathway of KIRC in the TCGA database. Table S1. Abbreviations for cancer in TCGA. Table S2. The relationship between clinical features and NCOA4 expression levels in COAD. Table S3. The relationship between clinical features and NCOA4 expression levels in KIRC. Table S4. The relationship between clinical features and NCOA4 expression levels in LIHC. Table S5. The relationship between clinical features and NCOA4 expression levels in BRCA. Table S6. The relationship between clinical features and NCOA4 expression levels in CESC. Table S7. The relationship between clinical features and NCOA4 expression levels in CHOL. Table S8. The relationship between clinical features and NCOA4 expression levels in LAML. Table S9. The relationship between clinical features and NCOA4 expression levels in LGG. Table S10. The relationship between clinical features and NCOA4 expression levels in PRAD. Table S11. The relationship between clinical features and NCOA4 expression levels in LUAD. Table S12. The relationship between clinical features and NCOA4 expression levels in LUSC. Table S13. The relationship between clinical features and NCOA4 expression levels in SARC. Table S14. The relationship between clinical features and NCOA4 expression levels in SKCM. [file 5242437.f1.zip › 5242437.f1/Supplementary Figures.pdf]

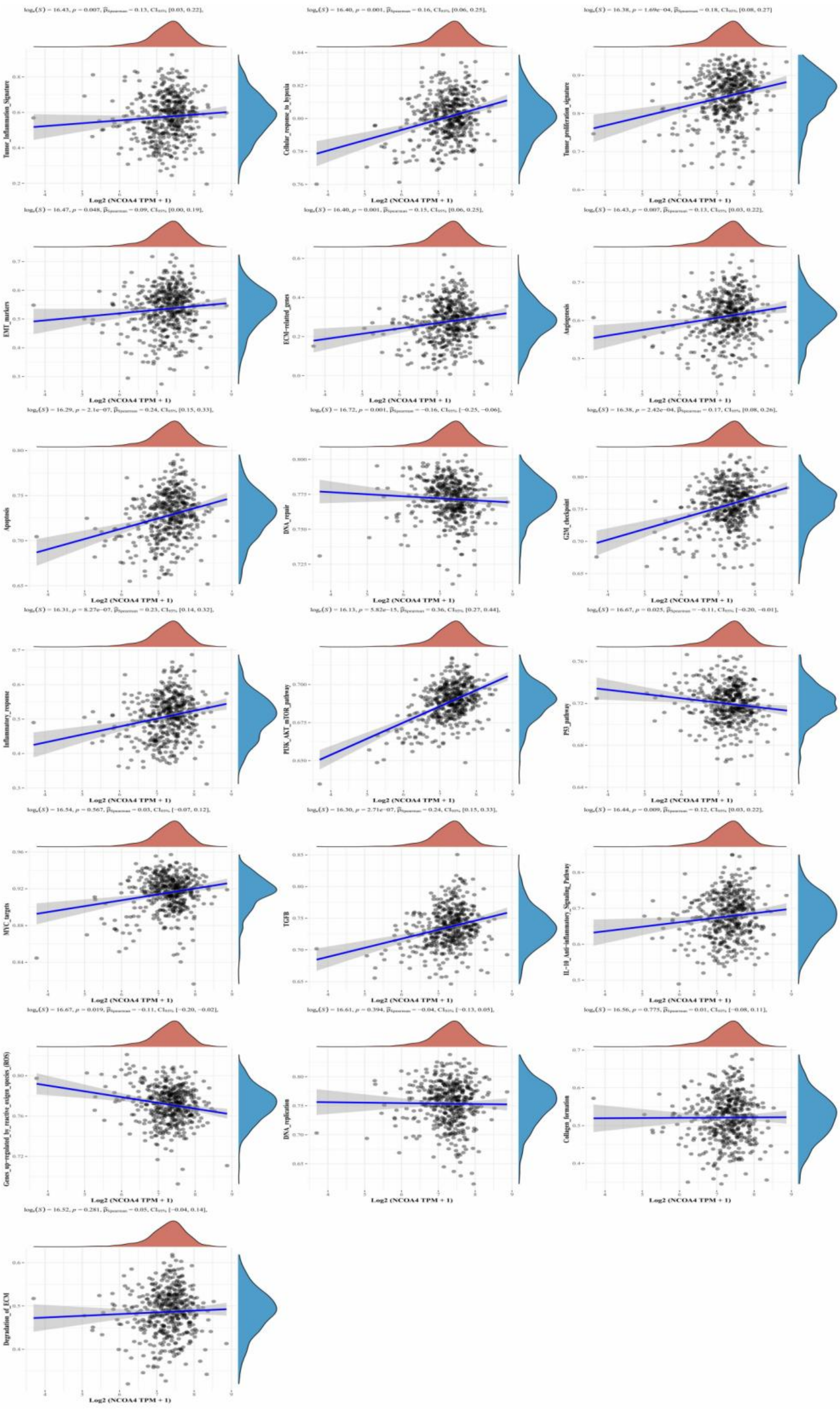

Figure S1. NCOA4-related genes hallmark enrichment pathway of COAD in the

TCGA database.

The relationship between NCOA4-related genes and traditional pathways, including Tumor_Inflammation_Signature, Cellular_response_to_hypoxia, Tumor_proliferation_signature, EMT_markers, ECM-relatted_genes, Angiogenesis, Apoptosis, DNA_repair, G2M_checkpoint, Inflammatory_response, PI3K_AKT_mTOR_pathway, P53_pathway, MYC_targets, TGFB, IL-10_Anti-inflammatory_Signaling_Pathway, Genes_up-regulated_by_reactive_oxigen_species_(ROS), DNA_replication, Collagen_formation, Degradation_of_ECM. The r range from -1 to 1, where $|r| \geq 0.7$ signifies a high positive correlation, $0.5 \leq |r| < 0.7$ is a moderate positive correlation, $0.3 \leq |r| <0.5$ is a low positive correlation, and $|r|< 0.3$ is a negligible correlation.

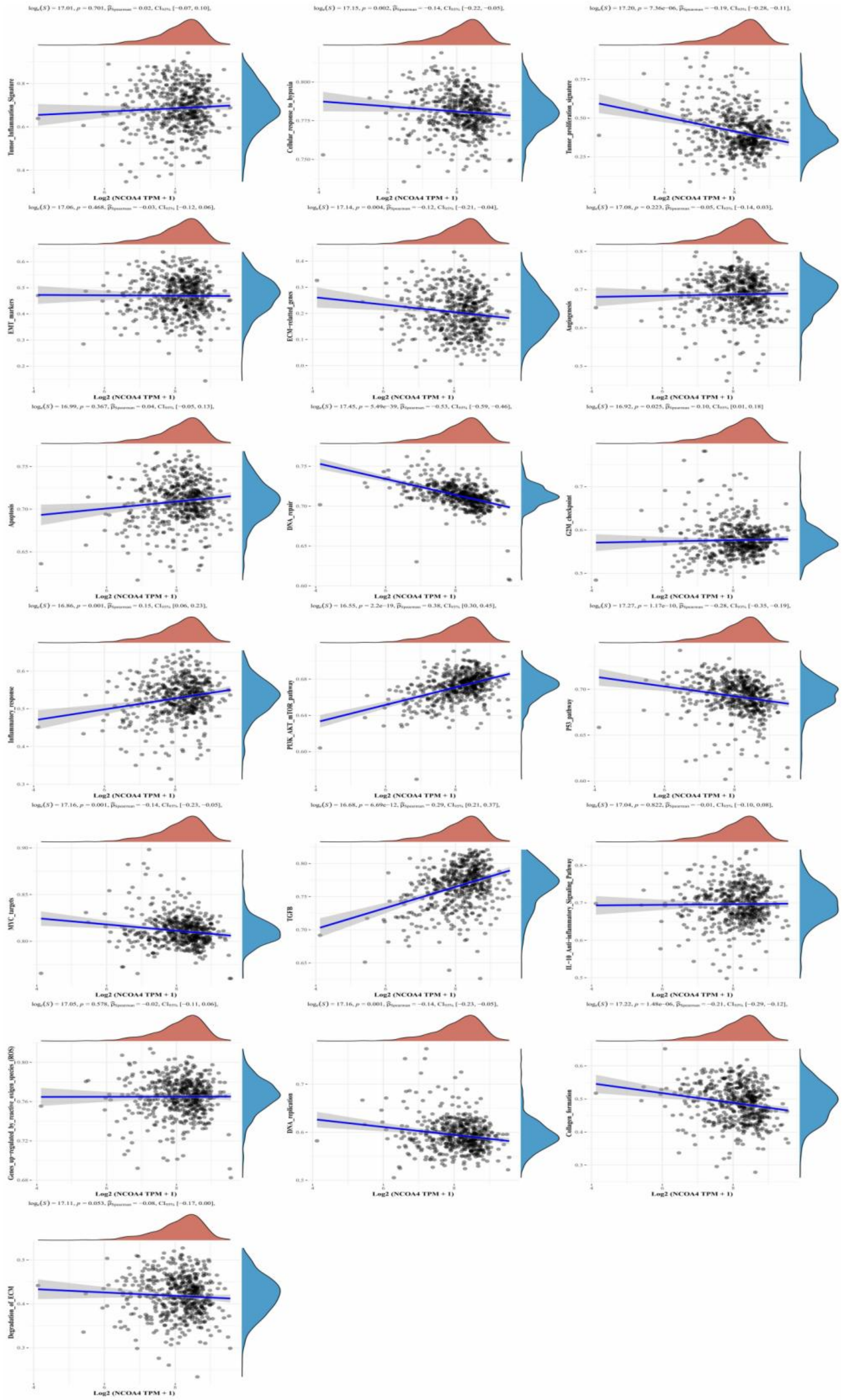

Figure S2. NCOA4-related genes hallmark enrichment pathway of KIRC in the

TCGA database.

Tumor_Inflammation_Signature, Cellular_response_to_hypoxia, Tumor_proliferation_signature, EMT_markers, ECM-relatted_genes, Angiogenesis, Apoptosis, DNA_repair, G2M_checkpoint, Inflammatory_response, PI3K_AKT_mTOR_pathway, P53_pathway, MYC_targets, TGFB, IL-10_Anti-inflammatory_Signaling_Pathway, Genes_up-regulated_by_reactive_oxigen_species_(ROS), DNA_replication, Collagen_formation, Degradation_of_ECM. The r range from -1 to 1, where $|r| \geq 0.7$ signifies a high positive correlation, $0.5 \leq |r| < 0.7$ is a moderate positive correlation, $0.3 \leq |r| < 0.5$ is a low positive correlation, and $|r| < 0.3$ is a negligible correlation.
