# Supplementary material for: NCOA4: An Immunomodulation-Related Prognostic Biomarker in Colon Adenocarcinoma and Pan-Cancer
Source: J Oncol. 2022 Jun 16;2022:5242437. doi: 10.1155/2022/5242437 (PMC9225891; doi:10.1155/2022/5242437)
Supplement: Supplementary Materials — Figure S1. NCOA4-related gene hallmark enrichment pathway of COAD in the TCGA database. Figure S2. NCOA4-related gene hallmark enrichment pathway of KIRC in the TCGA database. Table S1. Abbreviations for cancer in TCGA. Table S2. The relationship between clinical features and NCOA4 expression levels in COAD. Table S3. The relationship between clinical features and NCOA4 expression levels in KIRC. Table S4. The relationship between clinical features and NCOA4 expression levels in LIHC. Table S5. The relationship between clinical features and NCOA4 expression levels in BRCA. Table S6. The relationship between clinical features and NCOA4 expression levels in CESC. Table S7. The relationship between clinical features and NCOA4 expression levels in CHOL. Table S8. The relationship between clinical features and NCOA4 expression levels in LAML. Table S9. The relationship between clinical features and NCOA4 expression levels in LGG. Table S10. The relationship between clinical features and NCOA4 expression levels in PRAD. Table S11. The relationship between clinical features and NCOA4 expression levels in LUAD. Table S12. The relationship between clinical features and NCOA4 expression levels in LUSC. Table S13. The relationship between clinical features and NCOA4 expression levels in SARC. Table S14. The relationship between clinical features and NCOA4 expression levels in SKCM. [file 5242437.f1.zip › 5242437.f1/Supplementary Table1-14.pdf]

Table S1 Abbreviations for cancer in TCGA

Cancer type	Abbreviations
adrenocortical carcinoma	ACC
bladder urothelial carcinoma	BLCA
breast invasive carcinoma	BRCA
cervical squamous cell carcinoma	CESC
cholangiocarcinoma	CHOL
colon adenocarcinoma	COAD
lymphoid neoplasm diffuse large B cell lymphoma	DLBC
esophageal carcinoma	ESCA
glioblastoma	GBM
brain lower grade glioma	LGG
head and neck squamous cell carcinoma	HNSC
kidney chromophobe	KICH
kidney renal clear cell carcinoma	KIRC
kidney renal papillary cell carcinoma	KIRP
acute myeloid leukemia	LAML
liver hepatocellular carcinoma	LIHC
lung adenocarcinoma	LUAD
lung squamous cell carcinoma	LUSC
mesothelioma	MESO
ovarian serous cystadenocarcinoma	OV
pancreatic adenocarcinoma	PAAD
pheochromocytoma and paraganglioma	PCPG
prostate adenocarcinoma	PRAD
rectum adenocarcinoma	READ
sarcoma	SARC
skin cutaneous melanoma	SKCM
stomach adenocarcinoma	STAD
testicular germ cell tumors	TGCT
thyroid carcinoma	THCA
thymoma	THYM
uterine corpus endometrial carcinoma	UCEC
uterine carcinosarcoma	UCS
uveal melanoma	UVM

Table S2 The relationship between clinical features and NCOA4 expression levels in COAD

Characteristic	$NCOA4^{low}$	$NCOA4^{high}$	p
n	239	239	
Age, n (%)			1.000
<=65	97 (20.3%)	97 (20.3%)	
>65	142 (29.7%)	142 (29.7%)	
Gender, n (%)			0.647
Female	116 (24.3%)	110 (23%)	
Male	123 (25.7%)	129 (27%)	
Pathologic stage, n (%)			0.188
Stage I	40 (8.6%)	41 (8.8%)	
Stage II	83 (17.8%)	104 (22.3%)	
Stage III	72 (15.4%)	61 (13.1%)	
Stage IV	38 (8.1%)	28 (6%)	
T stage, n (%)			0.978
T1	5 (1%)	6 (1.3%)	
T2	41 (8.6%)	42 (8.8%)	
T3	163 (34.2%)	160 (33.5%)	
T4	29 (6.1%)	31 (6.5%)	
N stage, n (%)			0.126
N0	133 (27.8%)	151 (31.6%)	
N1	63 (13.2%)	45 (9.4%)	
N2	43 (9%)	43 (9%)	
M stage, n (%)			0.175
M0	166 (40%)	183 (44.1%)	
M1	38 (9.2%)	28 (6.7%)	
OS event, n (%)			0.002
Alive	173 (36.2%)	202 (42.3%)	
Dead	66 (13.8%)	37 (7.7%)	
DSS event, n (%)			0.027
Alive	186 (40.3%)	212 (45.9%)	
Dead	40 (8.7%)	24 (5.2%)	

Table S3 The relationship between clinical features and NCOA4 expression levels in KIRC

Characteristic	$NCOA4^{low}$	$NCOA4^{high}$	p
n	269	270	
Age, n (%)			0.322
<=60	128 (23.7%)	141 (26.2%)	
>60	141 (26.2%)	129 (23.9%)	
Gender, n (%)			0.002
Female	75 (13.9%)	111 (20.6%)	
Male	194 (36%)	159 (29.5%)	
Pathologic stage, n (%)			< 0.001
Stage I	114 (21.3%)	158 (29.5%)	
Stage II	30 (5.6%)	29 (5.4%)	
Stage III	68 (12.7%)	55 (10.3%)	
Stage IV	55 (10.3%)	27 (5%)	
T stage, n (%)			< 0.001
T1	116 (21.5%)	162 (30.1%)	
T2	40 (7.4%)	31 (5.8%)	
T3	104 (19.3%)	75 (13.9%)	
T4	9 (1.7%)	2 (0.4%)	
N stage, n (%)			1.000
N0	114 (44.4%)	127 (49.4%)	
N1	8 (3.1%)	8 (3.1%)	
M stage, n (%)			0.002
M0	195 (38.5%)	233 (46%)	
M1	51 (10.1%)	27 (5.3%)	
OS event, n (%)			< 0.001
Alive	153 (28.4%)	213 (39.5%)	
Dead	116 (21.5%)	57 (10.6%)	
DSS event, n (%)			< 0.001
Alive	183 (34.7%)	237 (44.9%)	
Dead	79 (15%)	29 (5.5%)	

Table S4 The relationship between clinical features and NCOA4 expression levels in LIHC

Characteristic	$NCOA4^{low}$	$NCOA4^{high}$	p
n	187	187	
Age, n (%)			0.043
<=60	78 (20.9%)	99 (26.5%)	
>60	108 (29%)	88 (23.6%)	
Gender, n (%)			0.507
Female	57 (15.2%)	64 (17.1%)	
Male	130 (34.8%)	123 (32.9%)	
Pathologic stage, n (%)			0.808
Stage I	89 (25.4%)	84 (24%)	
Stage II	43 (12.3%)	44 (12.6%)	
Stage III	39 (11.1%)	46 (13.1%)	
Stage IV	3 (0.9%)	2 (0.6%)	
T stage, n (%)			0.852
T1	92 (24.8%)	91 (24.5%)	
T2	46 (12.4%)	49 (13.2%)	
T3	40 (10.8%)	40 (10.8%)	
T4	8 (2.2%)	5 (1.3%)	
N stage, n (%)			0.125
N0	121 (46.9%)	133 (51.6%)	
N1	0 (0%)	4 (1.6%)	
M stage, n (%)			0.351
M0	127 (46.7%)	141 (51.8%)	
M1	3 (1.1%)	1 (0.4%)	
OS event, n (%)			0.745
Alive	120 (32.1%)	124 (33.2%)	
Dead	67 (17.9%)	63 (16.8%)	
DSS event, n (%)			0.956
Alive	142 (38.8%)	145 (39.6%)	
Dead	40 (10.9%)	39 (10.7%)	
Age, meidan (IQR)	64 (53, 69.75)	59 (51, 68)	0.020

Table S5 The relationship between clinical features and NCOA4 expression levels in BRCA

Characteristic	$NCOA4^{low}$	$NCOA4^{high}$	p
n	541	542	
Age, n (%)			0.411
<=60	293 (27.1%)	308 (28.4%)	
>60	248 (22.9%)	234 (21.6%)	
Pathologic stage, n (%)			0.834
Stage I	90 (8.5%)	91 (8.6%)	
Stage II	318 (30%)	301 (28.4%)	
Stage III	117 (11%)	125 (11.8%)	
Stage IV	10 (0.9%)	8 (0.8%)	
T stage, n (%)			0.357
T1	128 (11.9%)	149 (13.8%)	
T2	324 (30%)	305 (28.2%)	
T3	73 (6.8%)	66 (6.1%)	
T4	15 (1.4%)	20 (1.9%)	
N stage, n (%)			< 0.001
N0	286 (26.9%)	228 (21.4%)	
N1	153 (14.4%)	205 (19.3%)	
N2	51 (4.8%)	65 (6.1%)	
N3	42 (3.9%)	34 (3.2%)	
M stage, n (%)			0.658
M0	428 (46.4%)	474 (51.4%)	
M1	11 (1.2%)	9 (1%)	
OS event, n (%)			0.046
Alive	477 (44%)	454 (41.9%)	
Dead	64 (5.9%)	88 (8.1%)	
DSS event, n (%)			0.437
Alive	498 (46.8%)	480 (45.2%)	
Dead	39 (3.7%)	46 (4.3%)	
Age, meidan (IQR)	59 (49, 68)	58 (48, 67)	0.225

Table S6 The relationship between clinical features and NCOA4 expression levels in CESC

**Characteristic**	**NCOA4^{low}**	**NCOA4^{high}**	**p**
n	153	153	
Age, n (%)			0.003
<=50	81 (26.5%)	107 (35%)	
>50	72 (23.5%)	46 (15%)	
Clinical stage, n (%)			0.768
Stage I	78 (26.1%)	84 (28.1%)	
Stage II	33 (11%)	36 (12%)	
Stage III	24 (8%)	22 (7.4%)	
Stage IV	13 (4.3%)	9 (3%)	
T stage, n (%)			0.823
T1	67 (27.6%)	73 (30%)	
T2	38 (15.6%)	34 (14%)	
T3	11 (4.5%)	10 (4.1%)	
T4	6 (2.5%)	4 (1.6%)	
N stage, n (%)			1.000
N0	65 (33.3%)	69 (35.4%)	
N1	30 (15.4%)	31 (15.9%)	
M stage, n (%)			0.935
M0	60 (47.2%)	56 (44.1%)	
M1	5 (3.9%)	6 (4.7%)	
OS event, n (%)			0.345
Alive	121 (39.5%)	113 (36.9%)	
Dead	32 (10.5%)	40 (13.1%)	
DSS event, n (%)			0.551
Alive	126 (41.7%)	121 (40.1%)	
Dead	25 (8.3%)	30 (9.9%)	
Age, meidan (IQR)	49 (42, 60)	44 (35, 54)	< 0.001

Table S7 The relationship between clinical features and NCOA4 expression levels in CHOL

Characteristic	$NCOA4^{low}$	$NCOA4^{high}$	p
n	18	18	
Age, n (%)			0.505
<=65	7 (19.4%)	10 (27.8%)	
>65	11 (30.6%)	8 (22.2%)	
Gender, n (%)			1.000
Female	10 (27.8%)	10 (27.8%)	
Male	8 (22.2%)	8 (22.2%)	
Pathologic stage, n (%)			0.357
Stage I	9 (25%)	10 (27.8%)	
Stage II	3 (8.3%)	6 (16.7%)	
Stage III	1 (2.8%)	0 (0%)	
Stage IV	5 (13.9%)	2 (5.6%)	
T stage, n (%)			0.436
T1	9 (25%)	10 (27.8%)	
T2	5 (13.9%)	7 (19.4%)	
T3	4 (11.1%)	1 (2.8%)	
T4	0 (0%)	0 (0%)	
N stage, n (%)			0.636
N0	11 (35.5%)	15 (48.4%)	
N1	3 (9.7%)	2 (6.5%)	
M stage, n (%)			0.175
M0	12 (36.4%)	16 (48.5%)	
M1	4 (12.1%)	1 (3%)	
OS event, n (%)			0.002
Alive	4 (11.1%)	14 (38.9%)	
Dead	14 (38.9%)	4 (11.1%)	
DSS event, n (%)			0.007
Alive	5 (14.3%)	14 (40%)	
Dead	12 (34.3%)	4 (11.4%)	
Age, mean ± SD	66.44 ± 9.19	59.61 ± 15.19	0.112

Table S8 The relationship between clinical features and NCOA4 expression levels in LAML

Characteristic	$NCOA4^{low}$	$NCOA4^{high}$	p
n	75	76	
Age, n (%)			0.290
<=60	40 (26.5%)	48 (31.8%)	
>60	35 (23.2%)	28 (18.5%)	
Gender, n (%)			0.928
Female	33 (21.9%)	35 (23.2%)	
Male	42 (27.8%)	41 (27.2%)	
WBC count(x10^9/L), n (%)			0.072
<=20	44 (29.3%)	33 (22%)	
>20	30 (20%)	43 (28.7%)	
FLT3 mutation, n (%)			0.014
Negative	58 (39.5%)	44 (29.9%)	
Positive	15 (10.2%)	30 (20.4%)	
OS event, n (%)			0.431
Alive	24 (15.9%)	30 (19.9%)	
Dead	51 (33.8%)	46 (30.5%)	
Age, meidan (IQR)	58 (43, 71)	55.5 (42, 64)	0.204

Table S9 The relationship between clinical features and NCOA4 expression levels in LGG

**Characteristic**	**$NCOA4^{low}$**	**$NCOA4^{high}$**	**p**
n	264	264	
Age, n (%)			< 0.001
<=40	109 (20.6%)	155 (29.4%)	
>40	155 (29.4%)	109 (20.6%)	
Gender, n (%)			0.382
Female	125 (23.7%)	114 (21.6%)	
Male	139 (26.3%)	150 (28.4%)	
WHO grade, n (%)			0.638
G2	110 (23.6%)	114 (24.4%)	
G3	113 (24.2%)	130 (27.8%)	
OS event, n (%)			0.765
Alive	194 (36.7%)	198 (37.5%)	
Dead	70 (13.3%)	66 (12.5%)	
DSS event, n (%)			0.568
Alive	196 (37.7%)	201 (38.7%)	
Dead	65 (12.5%)	58 (11.2%)	
Age, meidan (IQR)	44 (34, 55)	38 (31, 49)	< 0.001

Table S10 The relationship between clinical features and NCOA4 expression levels in PRAD

**Characteristic**	**NCOA4^{low}**	**NCOA4^{high}**	**p**
n	249	250	
Age, n (%)			0.010
<=60	97 (19.4%)	127 (25.5%)	
>60	152 (30.5%)	123 (24.6%)	
T stage, n (%)			0.267
T2	85 (17.3%)	104 (21.1%)	
T3	153 (31.1%)	139 (28.3%)	
T4	6 (1.2%)	5 (1%)	
N stage, n (%)			0.152
N0	164 (38.5%)	183 (43%)	
N1	45 (10.6%)	34 (8%)	
M stage, n (%)			0.123
M0	225 (49.1%)	230 (50.2%)	
M1	3 (0.7%)	0 (0%)	
OS event, n (%)			0.544
Alive	243 (48.7%)	246 (49.3%)	
Dead	6 (1.2%)	4 (0.8%)	
DSS event, n (%)			0.686
Alive	245 (49.3%)	247 (49.7%)	
Dead	3 (0.6%)	2 (0.4%)	
Age, mean ± SD	62.09 ± 6.28	59.97 ± 7.17	< 0.001

Table S11 The relationship between clinical features and NCOA4 expression levels in LUAD

Characteristic	$NCOA4^{low}$	$NCOA4^{high}$	p
n	267	268	
Age, n (%)			0.134
<=65	138 (26.7%)	117 (22.7%)	
>65	123 (23.8%)	138 (26.7%)	
Gender, n (%)			0.631
Female	146 (27.3%)	140 (26.2%)	
Male	121 (22.6%)	128 (23.9%)	
Pathologic stage, n (%)			0.314
Stage I	144 (27.3%)	150 (28.5%)	
Stage II	67 (12.7%)	56 (10.6%)	
Stage III	42 (8%)	42 (8%)	
Stage IV	9 (1.7%)	17 (3.2%)	
T stage, n (%)			0.337
T1	94 (17.7%)	81 (15.2%)	
T2	138 (25.9%)	151 (28.4%)	
T3	27 (5.1%)	22 (4.1%)	
T4	7 (1.3%)	12 (2.3%)	
N stage, n (%)			0.807
N0	175 (33.7%)	173 (33.3%)	
N1	52 (10%)	43 (8.3%)	
N2	35 (6.7%)	39 (7.5%)	
N3	1 (0.2%)	1 (0.2%)	
M stage, n (%)			0.304
M0	176 (45.6%)	185 (47.9%)	
M1	9 (2.3%)	16 (4.1%)	
OS event, n (%)			0.022
Alive	158 (29.5%)	185 (34.6%)	
Dead	109 (20.4%)	83 (15.5%)	
DSS event, n (%)			0.484
Alive	183 (36.7%)	196 (39.3%)	
Dead	63 (12.6%)	57 (11.4%)	
Age, meidan (IQR)	65 (58, 72)	67 (59.5, 72.5)	0.122

Table S12 The relationship between clinical features and NCOA4 expression levels in LUSC

Characteristic	$NCOA4^{low}$	$NCOA4^{high}$	p
n	251	251	
Age, n (%)			0.825
<=65	94 (19.1%)	97 (19.7%)	
>65	153 (31%)	149 (30.2%)	
Gender, n (%)			0.155
Female	73 (14.5%)	58 (11.6%)	
Male	178 (35.5%)	193 (38.4%)	
Pathologic stage, n (%)			0.020
Stage I	118 (23.7%)	127 (25.5%)	
Stage II	94 (18.9%)	68 (13.7%)	
Stage III	36 (7.2%)	48 (9.6%)	
Stage IV	1 (0.2%)	6 (1.2%)	
T stage, n (%)			0.841
T1	53 (10.6%)	61 (12.2%)	
T2	151 (30.1%)	143 (28.5%)	
T3	36 (7.2%)	35 (7%)	
T4	11 (2.2%)	12 (2.4%)	
N stage, n (%)			0.348
N0	167 (33.7%)	153 (30.8%)	
N1	64 (12.9%)	67 (13.5%)	
N2	17 (3.4%)	23 (4.6%)	
N3	1 (0.2%)	4 (0.8%)	
M stage, n (%)			0.130
M0	192 (45.8%)	220 (52.5%)	
M1	1 (0.2%)	6 (1.4%)	
OS event, n (%)			0.321
Alive	149 (29.7%)	137 (27.3%)	
Dead	102 (20.3%)	114 (22.7%)	
DSS event, n (%)			0.567
Alive	184 (40.9%)	177 (39.3%)	
Dead	49 (10.9%)	40 (8.9%)	
Age, meidan (IQR)	69 (61, 73)	68 (62, 73)	0.609

Table S13 The relationship between clinical features and NCOA4 expression levels in SARC

Characteristic	$NCOA4^{low}$	$NCOA4^{high}$	p
n	131	132	
Age, n (%)			0.294
<=60	60 (22.8%)	70 (26.6%)	
>60	71 (27%)	62 (23.6%)	
Gender, n (%)			1.000
Female	72 (27.4%)	72 (27.4%)	
Male	59 (22.4%)	60 (22.8%)	
Histological type, n (%)			0.002
Dedifferentiated liposarcoma	19 (7.2%)	39 (14.8%)	
Desmoid Tumor	2 (0.8%)	0 (0%)	
Leiomyosarcoma	59 (22.4%)	47 (17.9%)	
Malignant Peripheral Nerve Sheath Tumors	4 (1.5%)	6 (2.3%)	
Myxofibrosarcoma	7 (2.7%)	18 (6.8%)	
Pleomorphic Sarcoma	34 (12.9%)	18 (6.8%)	
Synovial Sarcoma	6 (2.3%)	4 (1.5%)	
OS event, n (%)			0.115
Alive	75 (28.5%)	89 (33.8%)	
Dead	56 (21.3%)	43 (16.3%)	
DSS event, n (%)			0.107
Alive	82 (31.9%)	93 (36.2%)	
Dead	48 (18.7%)	34 (13.2%)	
Age, meidan (IQR)	63 (53, 73)	60 (52, 68)	0.341

Table S14 The relationship between clinical features and NCOA4 expression levels in SKCM

Characteristic	$NCOA4^{low}$	$NCOA4^{high}$	p
n	235	236	
Age, n (%)			0.885
<=60	127 (27.4%)	125 (27%)	
>60	104 (22.5%)	107 (23.1%)	
Gender, n (%)			1.000
Female	89 (18.9%)	90 (19.1%)	
Male	146 (31%)	146 (31%)	
Pathologic stage, n (%)			0.567
Stage I	35 (8.5%)	42 (10.2%)	
Stage II	73 (17.7%)	67 (16.3%)	
Stage III	93 (22.6%)	78 (18.9%)	
Stage IV	11 (2.7%)	13 (3.2%)	
T stage, n (%)			0.012
T1	24 (6.6%)	17 (4.7%)	
T2	31 (8.5%)	48 (13.2%)	
T3	41 (11.3%)	50 (13.7%)	
T4	91 (25%)	62 (17%)	
N stage, n (%)			0.607
N0	113 (27.3%)	122 (29.5%)	
N1	41 (9.9%)	33 (8%)	
N2	24 (5.8%)	25 (6%)	
N3	31 (7.5%)	25 (6%)	
M stage, n (%)			0.655
M0	212 (47.9%)	206 (46.5%)	
M1	11 (2.5%)	14 (3.2%)	
OS event, n (%)			0.005
Alive	139 (30%)	108 (23.3%)	
Dead	93 (20%)	124 (26.7%)	
DSS event, n (%)			0.008
Alive	148 (32.3%)	119 (26%)	
Dead	81 (17.7%)	110 (24%)	
Age, meidan (IQR)	58 (48, 71)	58 (47, 70.25)	0.751
